# Cocaine induces paradigm-specific changes to the transcriptome within the ventral tegmental area

**DOI:** 10.1038/s41386-021-01031-4

**Published:** 2021-06-21

**Authors:** Rianne R. Campbell, Siwei Chen, Joy H. Beardwood, Alberto J. López, Lilyana V. Pham, Ashley M. Keiser, Jessica E. Childs, Dina P. Matheos, Vivek Swarup, Pierre Baldi, Marcelo A. Wood

**Affiliations:** 1 Department of Neurobiology and Behavior, School of Biological Sciences University of California, Irvine, CA USA; 2 UC Irvine Center for Addiction Neuroscience, School of Biological Sciences, University of California, Irvine, CA USA; 3 Center for the Neurobiology of Learning and Memory, School of Biological Sciences, University of California, Irvine, CA USA; 4 Department of Computer Science, University of California, Irvine, CA USA; 5 Institute for Genomics and Bioinformatics, University of California, Irvine, CA USA; 6 Department of Pharmacology, Vanderbilt University School of Medicine, Nashville, TN USA

**Keywords:** Epigenetics and plasticity, Reward

## Abstract

During the initial stages of drug use, cocaine-induced neuroadaptations within the ventral tegmental area (VTA) are critical for drug-associated cue learning and drug reinforcement processes. These neuroadaptations occur, in part, from alterations to the transcriptome. Although cocaine-induced transcriptional mechanisms within the VTA have been examined, various regimens and paradigms have been employed to examine candidate target genes. In order to identify key genes and biological processes regulating cocaine-induced processes, we employed genome-wide RNA-sequencing to analyze transcriptional profiles within the VTA from male mice that underwent one of four commonly used paradigms: acute home cage injections of cocaine, chronic home cage injections of cocaine, cocaine-conditioning, or intravenous-self administration of cocaine. We found that cocaine alters distinct sets of VTA genes within each exposure paradigm. Using behavioral measures from cocaine self-administering mice, we also found several genes whose expression patterns corelate with cocaine intake. In addition to overall gene expression levels, we identified several predicted upstream regulators of cocaine-induced transcription shared across all paradigms. Although distinct gene sets were altered across cocaine exposure paradigms, we found, from Gene Ontology (GO) term analysis, that biological processes important for energy regulation and synaptic plasticity were affected across all cocaine paradigms. Coexpression analysis also identified gene networks that are altered by cocaine. These data indicate that cocaine alters networks enriched with glial cell markers of the VTA that are involved in gene regulation and synaptic processes. Our analyses demonstrate that transcriptional changes within the VTA depend on the route, dose and context of cocaine exposure, and highlight several biological processes affected by cocaine. Overall, these findings provide a unique resource of gene expression data for future studies examining novel cocaine gene targets that regulate drug-associated behaviors.

## Introduction

Drugs of abuse induce transcriptional changes within the reward circuitry that underlie persistent alterations in neuronal function and, ultimately, drug-seeking behavior [1–3]. In the initial stages of drug use, drug-induced neuroadaptions that occur within the ventral tegmental area (VTA) are thought to be critical for developing addiction-like behaviors [4, 5]. Studies investigating the underlying molecular mechanisms responsible for cocaine-induced VTA plasticity often focus on specific candidate genes [4–6]. Although these results identify various molecular mechanisms underlying cocaine-induced plasticity involved in the VTA, using a biased approach of selecting candidate genes can limit detection of the key biological processes that underlie cocaine-induced plasticity.

Throughout the addiction field, various cocaine exposure paradigms are employed to study specific drug-induced changes in neuroplasticity [7, 8]. These models range from home cage experimenter-delivered injections to sophisticated learning-based tasks [9, 10]. Often results are not compared across paradigms or may be unable to be compared due to variability in experimental designs [11–13]. Thus, it can be difficult to distinguish the fundamental mechanisms underlying a particular cocaine-related process.

Here, we characterized transcriptome-wide changes that occur within the VTA following one of the following cocaine exposure paradigms: acute home cage injections of cocaine/saline, chronic home cage injections of cocaine/saline, cocaine/saline-conditioning, or chronic intravenous-self administration of cocaine/saline. We discovered VTA transcriptome cocaine responses differ by paradigms with different doses, contexts, and delivery methods. However, our data revealed common biological processes that are affected across all cocaine paradigms. In addition, we found several common predicted upstream regulators present in all cocaine paradigms. Our results suggest that cocaine acts on various transcriptional networks of the VTA that are implicated in distinct aspects of drug-seeking behaviors. Our data also introduces novel transcriptional targets and biological processes to study in addiction-like behaviors and can serve as a dataset for future studies focused on mechanisms of cocaine-induced plasticity.

## Methods

### Mice

Adult (2 months–3 months) male C57BL/6J mice were used in these experiments with, with slight differences in age due to differences in exposure paradigms. Food and water were available ad libitum during 12:12 h light per dark cycle. All experiments were approved by the Institutional Animal Care and Use Committee of the University of California, Irvine. Subjects for each paradigm are the following: Acute home cage cocaine exposure (HC Acute Coc) (*n* = 6); acute saline HC exposure (HC Acute Sal) (*n* = 6); chronic cocaine home cage exposure (HC Chronic Coc) (*n* = 6); chronic saline HC exposure (HC Chronic Sal) (*n* = 6); acute cocaine contextual conditioning exposure (Conditioned Coc) (*n* = 6); acute saline contextual conditioning exposure (Conditioned Sal) (*n* = 6); chronic cocaine intravenous self-administration (IVSA Coc) (*n* = 15); chronic saline IVSA (IVSA Sal) (*n* = 8). Food and water were available ad libitum with lights on 12:12 h light per dark cycle.

### Home cage injections for gene expression analysis

Mice were given I.P. injections of either cocaine-HCl (20 mg/kg) or saline for either 1 day (HC Acute Coc or HC Acute Sal) or 7 days (HC Chronic Coc or HC Chronic Sal). One hour following the last injection, brain tissue was collected.

### Cocaine contextual conditioning exposure for conditioned gene expression analysis

Mice are confined to a novel chamber for 30 min following an injection of cocaine or saline (Conditioned Coc or Conditioned Sal). One hour following injection, brains were harvested for tissue processing.

### Cocaine-conditioned place preference (CPP)

CPP experiments were performed as described in previous studies (White et al. 2016; López et al. 2018). All mice underwent similar handling and pre-conditioning testing as described above. Using an unbiased paradigm, saline conditioned mice underwent two conditioning sessions where they received saline injections on both compartments. Mice that underwent one pairing conditioning received cocaine injections (i.p.; 20 mg/kg) prior to being placed in one context and on the following day, mice were injected with 0.9% saline before being placed in the alternate compartment. Mice that were subjected to two pairing conditioning underwent two cocaine and saline conditioning sessions in total, alternating between treatments each day. Twenty-four hours after the last conditioning session, preference (15 min, Posttest 1; day 8) was assessed in all animals similar to the pre-conditioning test in a drug-free state. CPP score was calculated as the time (s) spent in cocaine-paired minus saline-paired compartments. The EthoVision 3.1 software (Noldus Technology; see ref. [6]) was used to track time spent in each chamber of the CPP apparatus automatically from MPEG videos.

### Intravenous self administration

Mice were mildly food restricted to 85–90% of their free-feeding body weight and trained to press a lever in an operant chamber (Med Associates) for food pellets (20 mg; TestDiet) under a fixed-ratio 1, time out 20 s (FR1TO20 s) schedule of reinforcement (Supplementary Fig. 1). Once stable responding was achieved (>25 pellets per session across three subsequent sessions), the subjects were surgically catheterized. Mice were anesthetized with an isoflurane (1– 3%)/oxygen vapor mixture during surgery and implanted with intravenous catheters. The catheter tubing was passed subcutaneously into the jugular vein, following the surgery, animals recovered for ≥48 h prior to self-administration. Subjects were then permitted to acquire intravenous cocaine self-administration (IVSA) during 1 h daily sessions for 7 consecutive days. Cocaine was delivered through the intravenous catheter by a Razel syringe pump (Med Associates). Each session was performed using two retractable levers (1 active, 1 inactive). Completion of the response criteria on the active lever resulted in the delivery of an intravenous cocaine infusion (0.03 ml infusion volume; FR1TO20 s schedule) at a dose of 0.5 mg/kg/infusion. Responses on the inactive lever were recorded but had no scheduled consequences. Catheters were flushed daily with physiological sterile saline solution (0.9% w/v) containing heparin (100 USP U/ml). Subjects and their data were removed from the study if the catheter integrity was compromised as determined by visual leakage or intravenous propofol assessment (propofol sodium, Patterson Vet). Behavioral responses were automatically recorded by Med Associates software. Tissue was collected 1 h following the end of the last session. Groups are referred to as ‘IVSA Coc’ or ‘IVSA Sal’.

### Drugs

Cocaine-HCl (Sigma-Aldrich: St. Louis, Missouri, USA) was dissolved in saline (0.9% NaCl). For non-contingent exposure paradigms, intraperitoneal injections of 20 mg/kg cocaine were given. For IVSA (FR1TO20 s schedule) at a dose of 0.5 mg/kg/infusion during 1 h daily sessions for 7 consecutive days. Dose was selected based off previous work from the field [14–16].

### Tissue collection

Mice were sacrificed 1 h following either the last injection or IVSA session and brains were flash frozen. This time point was selected based on previous studies showing robust changes in gene expression following either a learning or cocaine-related event [17–19]. 1 mm punches from coronal sections of both hemispheres were collected from 500 μm slices of the VTA (Allen Brain atlas coordinates (−3.30 mm to −2.80 mm from Bregma).

### RNA sequencing

RNA was isolated from VTA punches using the RNeasy minikit (Qiagen, 74104). RNA quality was assessed by Bioanalyzer. Samples with an RNA integrity number >9 were used. cDNA libraries were prepared with starting 100 ng of total RNA using the TruSeq RNA Sample Preparation Kit (Illumina). The quality of the remaining sequences was further assessed using PHRED quality scores produced in real time during the base-calling step of the sequencing run (Supplementary Fig. 2).

### Gene expression and differential analysis

FastQ files are processed through standard Tuxedo protocol outputting FPKM values for each gene of each replicate. Differential analysis of gene expression is conducted with Cyber-T, an analysis program using Bayesian-regularized *t*-test (Supplementary Table 1). For comparison, differential analysis was also conducted using linear regression models as previously described [20, 21] (Supplementary Table 1). Genes were first ranked by uncorrected *p* value, and only genes with both uncorrected *p* value < 0.05, and fold-changes > |0.5| were used for analysis. Fisher’s exact tests (FET) were conducted to compare DEGs across paradigms. Top up- or downregulated genes are identified for further analysis.

*A detailed description of methods is provided in the**Supplementary Methods section*.

## Results

### VTA transcriptional patterns induced by cocaine depend on the exposure paradigm

**Fig. 1:**
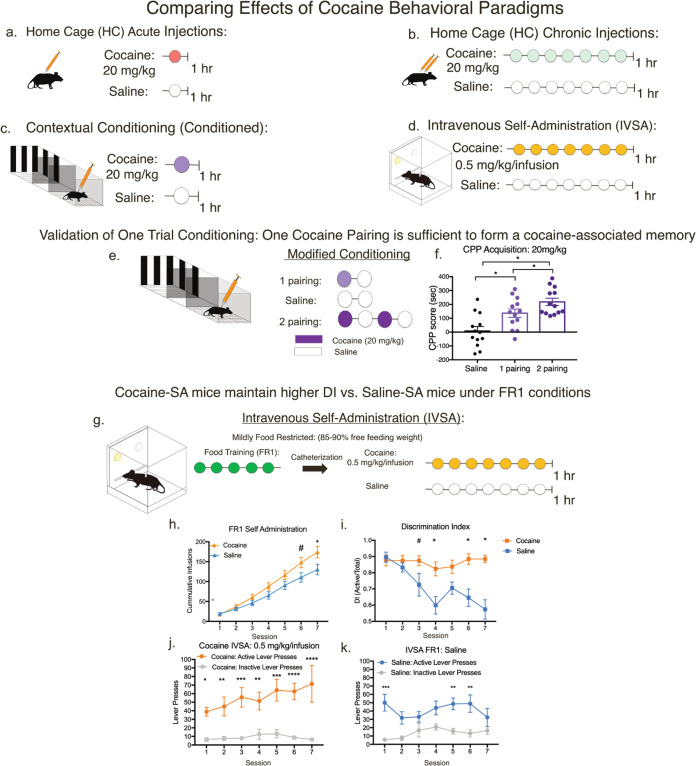
Conventional cocaine behavioral paradigms used to identify paradigm-specific changes to the VTA Transcriptome. **a** Schematic timeline of home cage injections. Mice either underwent acute I.P. injections with cocaine (20 mg/kg) or saline (HC Acute) or were (**b**) chronic I.P. injections with cocaine or saline (HC Chronic). Tissue was collected 1 h following the last injection. **c** Schematic timeline of animals undergoing modified contextual conditioning. Mice underwent one conditioning session of either cocaine (20 mg/kg) or saline (Conditioned). Tissue was collected 1 h following the I.P. injection of either cocaine of saline. **d** Schematic of IVSA paradigm. Mice were mildly food restricted (85–90% free-feeding body weight) and following food training at an FR1 schedule (Supplementary Fig. 1), mice underwent either 7 days of IVSA cocaine or saline. Tissue was collect 1 h following completion of the last IVSA session. **e**, **f** Cocaine-associated Memories are formed within One Cocaine Contextual Conditioning Session and Acquisition of Cocaine-self-administration occurs within 1 Week of IVSA In Adult Male Mice. **e** Mice underwent either one of the following using contextual conditioning boxes: one conditioning session of cocaine and saline; two conditioning sessions of cocaine and saline; or four conditioning sessions of only saline. **f** Conditioning with either one pairing or two pairings of cocaine and saline induced a higher contextual place preference score than saline alone (One-way ANOVA with Tukey’s multiple comparisons *p < 0.05, **p < 0.0001). **g**–**k** Cocaine is reinforcing under FR1 conditions. **g** Schematic of IVSA paradigm. While mildly food restricted, mice underwent food training at a FR1 schedule and subsequently underwent either 7 days of IVSA cocaine or saline. **h** Cumulative record of cocaine or saline infusions of mice. (Two-way ANOVA repeated measure Cocaine effect (*F*1,21) = 2.784, *p* = 0.112; Session effect *F*(6,126) = 134.5, *p* < 0.0001; Interaction effect *F*(6,126) = 3.634, *p* = 0.0023). Mice self-administered for either cocaine or saline across 7 days. **i**. Cocaine self-administering mice discriminate more between active levers than saline (Two-way ANOVA repeated measure: Session effect *F*(6,126) = 3.235, *p* = 0.005; Cocaine effect *F*(1,21) = 25.67, *p* < 0.001; Interaction effect *F*(6,126) = 2.556, *p* = 0.0227). **j** Mice with access to IV cocaine underwent reinforcement learning, shown by the discrimination between active lever pressing vs. the inactive lever (Two-way ANOVA repeated measure Session effect (*F*1,14) = 52.98, *p* = 0.3224; Lever effect *F*(6, 1,14) = 52.98, *p* < 0.0001; Interaction effect *F*(6,84) = 0.9885, *p* = 0.4384). **k** Mice with access to IV saline failed to discriminate between levers (Two-way ANOVA repeated measure Lever effect (*F*1,14) = 25.88, *p* = 0.0002; Session effect *F*(6,84) = 1.144, *p* = 0.3442; Interaction effect *F*(6,84) = 0.6, *p* = 0.2165).

To investigate how a particular cocaine exposure paradigm affects the VTA transcriptome, we performed RNA-sequencing from whole VTA tissue punches of adult male mice that underwent one of the following eight paradigms: acute home cage cocaine exposure (HC Acute Coc; *n* = 6); acute saline home cage exposure (HC Acute Sal; *n* = 6); chronic cocaine home cage exposure (HC Chronic Coc; *n* = 6); chronic saline home cage exposure (HC Chronic Sal; *n* = 6); acute cocaine contextual conditioning exposure (Conditioned Coc); acute saline contextual conditioning exposure (Conditioned Sal; *n* = 6); chronic cocaine IVSA (IVSA Coc; *n* = 15); and chronic saline IVSA (IVSA Sal; *n* = 8) (Fig. 1a-d).

First, we validated the behavioral approaches. For the Conditioned groups, an adapted one-trial session of conditioned place preference model was used to understand how cocaine-associated learning affects VTA gene expression. This was validated using a separate cohort of mice that underwent one of the following using contextual conditioning boxes: one conditioning session of cocaine and saline; two conditioning sessions of cocaine and saline; or four conditioning sessions of only saline (Fig. 1e). Conditioning with either one pairing or two pairings of cocaine and saline produced a higher contextual place preference score than saline alone (Fig. 1f: One-way ANOVA with Tukey’s multiple comparisons *p < 0.05, **p < 0.0001). This demonstrates that one conditioning session is sufficient for mice to acquire a place preference.

For the IVSA groups, VTA tissue was collected from mice that were mildly food restricted and food trained (Supplementary Fig 1) at an FR1 schedule prior to 7 days of IVSA cocaine or saline (Fig. 1g). Cocaine IVSA mice show significantly more cumulative infusions than saline IVSA mice on the last IVSA sessions (Fig. 1h:Two-way ANOVA repeated measure: Session effect: *p* < 0.0001; Interaction effect: *p* = 0.0023) and discriminate significantly more between levers than saline IVSA mice (Fig. 1i.: Two-way ANOVA repeated measure: Session effect: *p* = 0.005; Cocaine effect: *p* < 0.001; Interaction effect: *p* = 0.0227; Fig. 1j.: Two-way ANOVA repeated measure: Lever effect: *p* < 0.0001); Fig. 1k: Two-way ANOVA repeated measure: Lever effect: *p* = 0.0002). Thus, although IVSA saline mice lever press for saline, cocaine IVSA mice have learned to perform the operant task and discriminate for the active lever for cocaine delivery across sessions. Together, the data shown in Fig. 1 validate the behavioral approaches used to induce cocaine-associated gene expression.

**Fig. 2:**
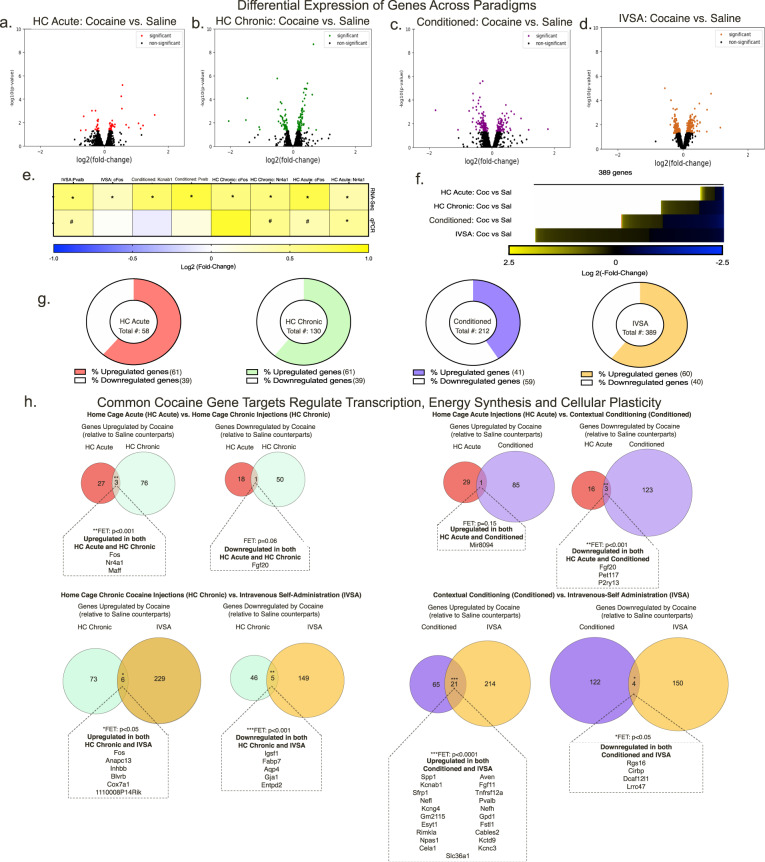
Cocaine alters gene expression within the VTA in a paradigm-specific manner. **a**–**d** Volcano plots illustrating significance (*Y*-axis) and magnitude (*X*-axis) of cocaine-induced changes from each paradigm. **e** Expression changes for a semi-random subset of genes was analyzed by qPCR for each paradigm from RNA-Seq samples. Cocaine-induced expression change is represented by Log2(fold-change) vs. saline group from each paradigm and compare to the fold change found by RNA-Seq. * = *p* < 0.05; ^#^ = *p* < 0.1. **f** Heat map comparing number and magnitude of cocaine-induced gene expression changes in each group.. **g** Charts showing the percentage of upregulated and downregulated genes by cocaine from each paradigm and listed total number of differentially expressed genes (genes that were up- and downregulated from each cocaine-treated group relative are relative to the saline counterparts within each paradigm (FC < 0.5, uncorrected p value < 0.05). **h** Cocaine induces paradigm-specific changes in gene expression within the VTA. All Venn Diagrams show upregulated or downregulated genes (FC > 0.5, *p* < 0.05) by cocaine relative to the saline controls within that specific paradigm. Any genes upregulated or downregulated by both paradigms being compared in Venn Diagram are listed. Number of common and/or distinct upregulated DEGS and downregulated DEGs (FC, 0.5 *p* < 0.05) relative to saline-controls from each paradigm shown with Venn Diagrams.

Next, we examined how each paradigm affects broad patterns of cocaine-induced gene expression by comparing each cocaine group to the appropriate saline control (Fig. 2a-d; Supplementary Fig. 2; Supplementary Data Table 1). We also validated several DEGs using RT-qPCR (Fig. 2e). Overall, we observed that the number of differentially expressed genes (DEGs) relates to the cocaine exposure history and the type of cocaine administration (Fig. 2f). In addition, all experimenter-injected cocaine exposure paradigms exhibit similar log_2_(FC). The IVSA cocaine induces a greater number of DEGs compared to the other paradigms, however DEGs within IVSA exhibit lower log-fold changes than the other paradigms. In terms of the direction of changes in gene expression, cocaine overall upregulates a higher percentage of genes in the VTA across all paradigms, except for the Conditioned paradigm (Fig. 2g). These data suggest that the paradigm can influence the directionality and overall number of DEGs. From our datasets we also found several genes implicated in cocaine action based on previous literature. From HC Acute cocaine exposure, we found upregulation of the immediate-early genes (IEGs) *Nr4a1* and *Fos* [14, 22–26]. Repeated home cage cocaine exposure (HC Chronic) upregulated expression of several genes that are increased within the midbrain of human chronic cocaine users (*Cdnk1a, Nfkbia* and *Fosb)* [27] or by chronic cocaine in preclinical mouse studies (*Fosb*) [24, 28]. Genes altered by Conditioned Cocaine include *Nr4a2* and *Bdnf* [29–31]. In the IVSA paradigm, several circadian genes *(Per1/2/3, Cry1/2)* and IEGs (*Egr1*, *Jun*) were altered by cocaine [32–34]. C1ql2 was also upregulated following IVSA, which is associated with human cocaine addiction using GWAS [35]. IVSA cocaine also increased expression of *Cartpt* and decreased expression of *Mef2a*, both genes previously studied in cocaine action [15, 22]. When comparing DEGs in our IVSA paradigm to two recent RNA-sequencing studies [3, 22], we detected several overlapping DEGs (Supplementary Data Table 2). This includes known gene targets such as *Fos, Cartpt, Egr1, Per1* as well as novel cocaine targets (e.g., *Fgf10*, *Trem2*).

### Common DEGs altered by cocaine participate in transcriptional regulation, energy synthesis, and cellular plasticity

After identifying DEGs within each paradigm, we next examined DEGs that were upregulated or downregulated in comparing one cocaine exposure paradigm to another using FET and rank-rank hypergeometric overlap. Little overlap in DEGs was found between the cocaine exposure paradigms (Fig. 2h; Supplementary Fig. 3). This further supports the idea that cocaine differentially affects molecular mechanisms based on the context, dose and route of administration.

We detected a small number of DEGs common across multiple paradigms that have also been identified in previous studies (Supplementary Fig. 4). For example, when comparing HC Acute vs. HC Chronic (FET: *p* < 0.001), two common DEGs are the CREB-regulated IEGs: *Fos* and *Nr4a1*. *Fos* expression was also upregulated by both IVSA and HC Chronic cocaine (FET: *p* < 0.05) as observed in previous studies [36, 37]. Several IEGs altered in at least one of our cocaine exposure paradigms were also dysregulated in human tissue of cocaine abusers (HC Chronic: *Cdk1a*, *Fosb*, and *NfkBia*; IVSA: *Egr1* and *Jun*) [27, 38]. The altered expression of these IEGs in chronic cocaine exposure paradigms and in human cocaine abusers suggest they may mediate persistent cocaine-induced changes within the VTA. Overall, given the consistent patterns of expression across paradigms and studies, these immediate-early genes may be key in regulating cocaine action in the VTA.

We also uncovered novel cocaine targets affected similarly across exposure paradigms. In the HC exposure paradigms (HC Acute vs. HC Chronic), the CREB-regulated gene *Maff* [39] was upregulated in both cocaine paradigms. Several DEGs identified across paradigms are known to regulate neuronal activity and plasticity, but have not yet been studied in cocaine action. For example, in the HC Chronic, HC Acute, and Conditioned paradigms, the neurotrophic factor *Fgf20* [40] was downregulated by cocaine. *P2ry13* [41], a microglia-specific gene was also downregulated by acute cocaine exposure paradigms (HC Acute vs. Conditioned; FET: *p* < 0.001). Genes related to energy regulation were also altered by cocaine across several paradigms. This includes *Pet117*, which was downregulated by HC Acute and Conditioned cocaine. In addition, the mitochondrial genes *Cox7a1 and Entpd2* were altered by both the IVSA and HC Chronic paradigms (FET: *p* < 0.001). From this analysis, these altered gene targets suggest mechanisms related to neuroprotection and energy synthesis within the VTA are dysregulated by cocaine.

When comparing genes altered in both Conditioned and IVSA cocaine groups, we found several DEGs implicated in synaptic function (upregulated: FET: *p* < 0.0001; downregulated: FET: *p* < 0.05). This includes genes encoding potassium channels (*Kcnab1, Kcng4, Kctd9, Kcnc3)* that were upregulated from both cocaine paradigms. In addition, we found upregulation of Nef/neurofilament-related genes (Nefl, Nefh) in both Conditioned and IVSA paradigms. Overall, these newly identified DEGs shared across cocaine exposure paradigms serve as potential targets for studying cocaine-induced plasticity.

### Cocaine alters processes important for cellular responses to stimuli and mitochondrial function across cocaine exposure paradigms

**Fig. 3:**
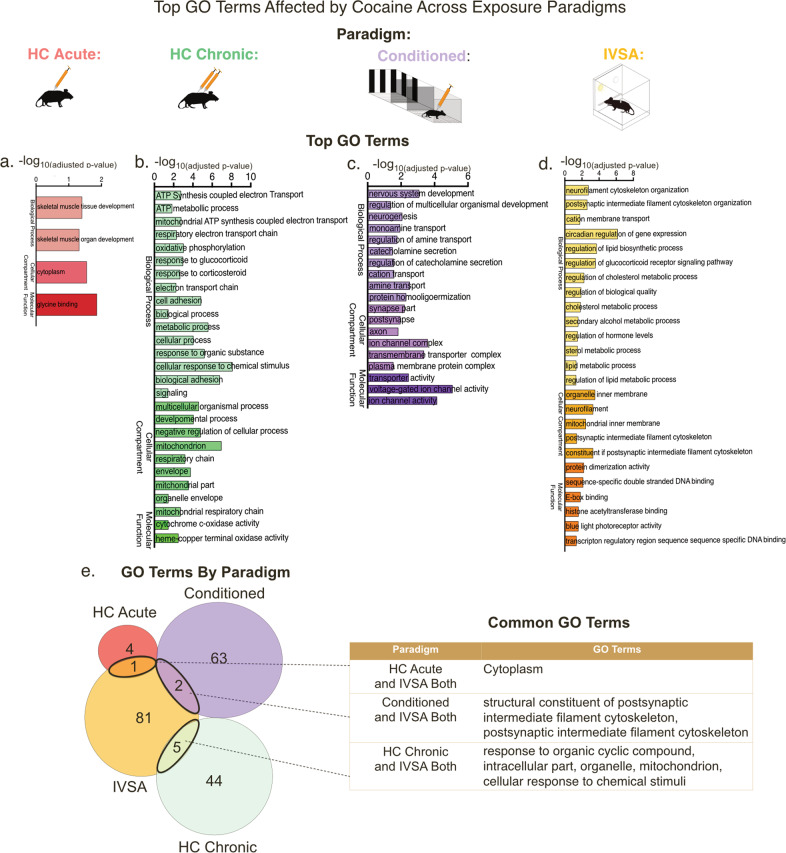
Predicted biological processes altered by each cocaine exposure paradigm. **a**–**d** Top gene ontology (GO) terms of both upregulated and downregulated DEGs within each paradigm are listed. **e** Common GO terms affected by cocaine across paradigms.

Using Gene ontology (GO) term analysis, we next identified key biological processes associated with each cocaine exposure paradigm. The HC Acute cocaine exposure group revealed few biological processes (skeletal muscle tissue development; glycine binding) (Fig. 3a). In contrast, the HC Chronic group contained numerous biological processes including ATP synthesis and metabolism, hormonal response to glucocorticoids and corticosteroids, and several mitochondrional processes (Fig. 3b). Altered ATP metabolic processes have also been seen within human tissue of cocaine abusers [38]. Thus, repeated cocaine exposure may recruit and dysregulate more biological functions within the VTA than one initial exposure. In addition, in comparison to acute cocaine, chronic cocaine-induced changes in gene expression may be affecting processes that lead to persistent changes in cellular function, such as energy regulation.

Conditioned cocaine DEGs were associated with distinct cellular processes including neurogenesis, transporter and ion channel activity (Fig. 3c) [42–44]. Similar to the other paradigms, we found that the IVSA group contained processes related to transporter activity, glucocorticoid receptor responses, cytoskeleton organization, and metabolism/mitochondrial functions (Fig. 3d). Cocaine-altered biological processes specific to IVSA included processes relating to circadian rhythms and histone acetyltransferase binding (Fig. 3d). This analysis overall illustrates the distinct functions that may be altered in each paradigm and suggests possible key mechanisms for further investigation.

Common GO terms between paradigms were identified using Venn diagram comparisons (Fig. 3e). We found little overlap of associated biological processes between groups, similar to the gene expression analysis. Following these comparisons, we identified biological processes that were associated with more than one cocaine exposure paradigm. We found two GO terms related to the synapse, the structural constituent of post synaptic intermediate filament skeleton and the post synaptic intermediate filament cytoskeleton, that were affected by both Conditioned cocaine and IVSA cocaine. Two genes under these GO terms (*Nefh, Nefl*) were altered in a similar direction in both paradigms (Supplementary Fig. 5). Common biological processes between the HC Chronic and IVSA groups include response to organic cyclic compound, intracellular part, organelle, mitochondrion, cellular response to chemical stimuli. When examining the mitochondrian GO-term, we find that although there are common genes within each list (i.e., *Cox7a1*, *Ndufa2*), again the majority of DEGs are unshared (Supplementary Fig. 5). Overall, our findings suggest that these common biological processes are important to cocaine action, however genes that are dysregulated by cocaine to promote changes in these potential biological processes are specific to a particular exposure paradigm.

### Predicted upstream regulators of cocaine-induced gene expression within the VTA across all exposure paradigms

**Fig. 4:**
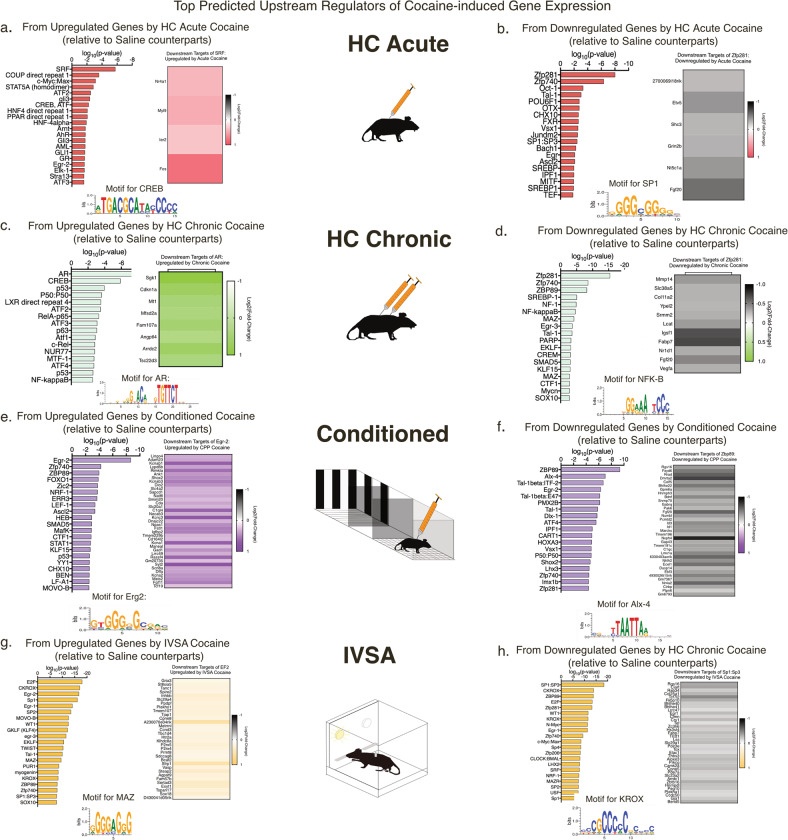
Top predicted upstream regulators of both upregulated genes and downregulated genes induced by cocaine in each paradigm. **a**–**h** Top predicted upstream regulators of differentially expressed genes by each cocaine exposure paradigm. Heat maps show the log-fold changes of the genes targeted by the top upstream regulator from each cocaine exposure paradigm. Below each comparison is a DNA motif of the binding sequences of a top upstream regulator in a specific paradigm.

We next sought to identify the upstream transcription factors (TFs) that regulate paradigm-specific changes in gene expression. Figure 4a-h lists the top predicted upstream regulators determined by MotifMap analysis for the genes upregulated or downregulated by cocaine within each paradigm. In HC Acute and HC Chronic paradigms, ZFP281 is the top common regulator for genes downregulated by cocaine (Fig. 4b,d). *Fgf20* is the only shared gene target from both paradigms, illustrating that ZFP281 targets distinct genes within each paradigm (Fig. 4b,d). This result sheds light on two mechanisms relating to cocaine action: (1) the importance in studying the relationship between ZFP281 and *Fgf20* and (2) cocaine may alter key TFs that, through coordinated actions with other transcription complexes, regulate the expression of genes critical for plasticity related to a specific cocaine experience.

To investigate potential regulators of cocaine-induced gene expression, we compared the overlap of the top 30 (determined by *p* value) upstream regulators of cocaine-induced gene expression for each paradigm (Supplementary Fig. 6). Among the common TFs targeting genes upregulated by cocaine, we identified several previously identified regulators of cocaine-induced gene expression (e.g., CREB, SRF, ATF(1/2/3) and EGR2/3)(Supplementary Fig. 6a) [16, 45–47]. Several of these TFs were also predicted upstream regulators of the genes downregulated by cocaine: EGR-2, CART and NF-KB (Supplementary Fig. 6b) [48–50]. This indicates that cocaine alters the function of transcription regulators in a wide-scale fashion, regulating expression of both up- and downregulated genes. Novel regulators of cocaine-induced gene expression were also detected: ZFP40, ZBP89, SP1/2, and MAZ. In addition, the two common TFs across all cocaine exposure paradigms have yet to be studied within the addiction field: c-Myc:Max and RFX1. These findings suggest that these TFs may participate in coordinated regulation of cocaine-induced gene expression in the VTA.

### Gene expression levels correlating with individual patterns of cocaine infusions with cocaine IVSA

**Fig. 5:**
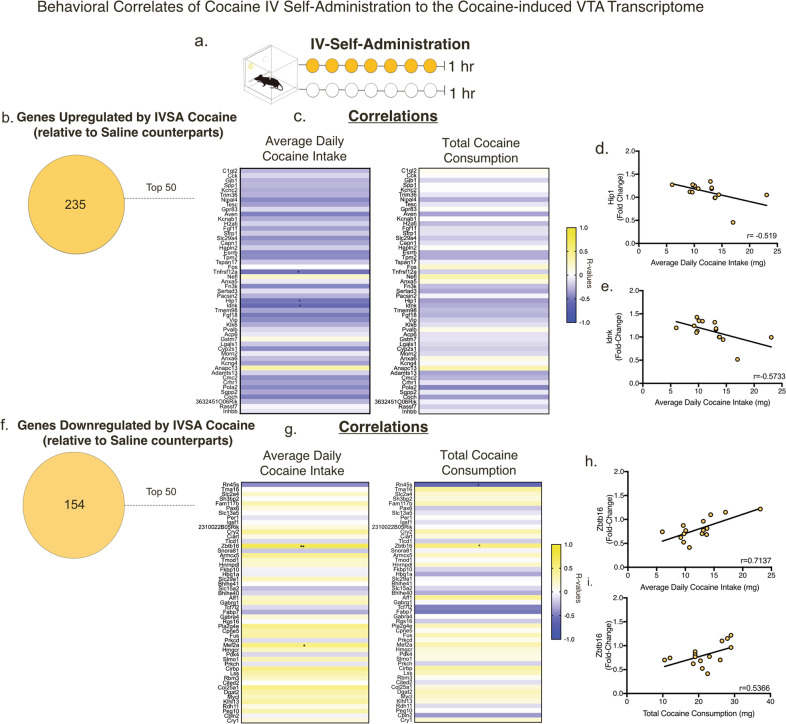
Gene expression levels correlating with one behavioral measure of cocaine IVSA. **a** Schematic of IVSA. **b** Number of genes upregulated in the VTA by cocaine self-administration compared to saline self-administration. **c** Correlation heat map of the fold changes from the top 50 genes with the average daily cocaine intake (Tnfrsf12a, Hip1, Idnk) (left) and total cocaine consumption (right). Exact *r* values for each gene and exact *p* values are available in Supplementary Data 2. **d** Fold changes of Idnk correlate with average daily cocaine intake (*r* = 0.5437, *p* = 0.03). **e** Number of genes downregulated in the VTA by cocaine self-administration compared to saline self-administration. **f** Correlation heat map of the fold changes from the top 50 genes with the average daily cocaine intake (Zbtb16, Mef2a) (left) and total cocaine consumption (Rn45s, Zbtb16) (right). **g** Zbtb16 fold changes correlate with average daily cocaine intake (*r* = 0.7137, *p* = 0.002) and (**h**) total cocaine consumption (*r* = 0.5366, *p* = 0.03). (* = *p* < 0.05, ** = *p* < 0.01).

To further our analyses, we sought to identify genes that mediate processes specific to cocaine reinforcement and behavioral responses to cocaine by correlating the fold changes of the top 50 (determined by *p* value) upregulated and downregulated genes (*p* < 0.05) in the IVSA groups with two different behavioral factors: average daily cocaine intake and total cocaine consumption during all IVSA behavioral sessions. A heat map of Pearson’s *r* values is presented in Fig. 5 (exact r and *p* values for all targets are available in Supplementary Data Table 3. In the upregulated gene sets (Fig. 5b), the expression profiles of three genes negatively correlated with average daily cocaine intake (*Tnfrsf12a, Hip1, Idnk*) whereas, zero genes correlated with total cocaine consumption (Fig. 5c-e).

From the top 50 genes downregulated by cocaine (Fig. 5f), two genes positively correlated with average daily cocaine intake *(Zbtb16, Mef2a)* and fold changes of 2 genes (*Rn45s, Zbtb16*) correlated with total cocaine consumption (Fig. 5g-i). Overall, this analysis suggests that these genes may have important involvement in processes underlying cocaine reinforcement.

### Coexpression analysis identifies gene networks altered by cocaine implicated in gene regulation and synaptic processes within specific cell types of the VTA

**Fig. 6:**
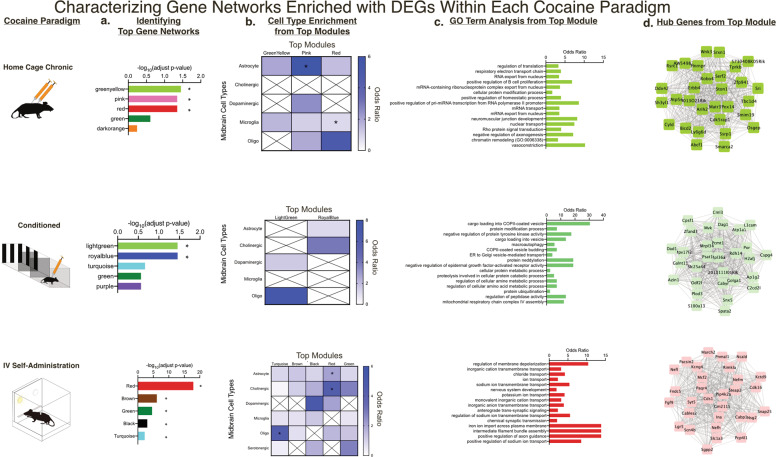
Coexpression analysis identifies gene networks altered by cocaine implicated in gene regulation and synaptic processes within specific cell types of the VTA. **a** WGCNA identified gene coexpression relationships, formed into modules, within each cocaine exposure paradigm. The modules with significant enrichment (* = *p* < 0.05) after Bonferroni correction are plotted. **b** Cell type enrichment analysis on the top modules shows that gene networks are altered within various cell types, primarily glial cells. **c** GO term analysis conducted on the top module in each paradigm shows that gene networks are altered within many biological processes, including those related to synaptic processes and gene regulation. **d** 30 most connected “hub” genes are identified from the top module in each paradigm.

Weighted gene coexpression analysis (WGCNA) was performed to identify gene coexpression relationships within each cocaine exposure paradigm (Supplementary Fig. 7). Modules overrepresented with DEGs and with highest significant enrichment after FDR correction are plotted (Fig. 6a). Note that although some module color names are reused within each network, these modules are completely independent with no similarity of gene members in each module. We found that the chronic exposure paradigms had more modules survive correction in comparison to the others, including HC Acute which had no modules survive. This again suggests that VTA transcriptome responses differ by paradigms with different doses, context and route of cocaine administration.

Cell type enrichment analysis was next performed on the enriched modules within each paradigm to understand how these gene networks alter VTA activity (Fig. 6b). HC Chronic and IVSA had enriched glial markers, which may signify the importance of these cell types within the VTA following repeated cocaine exposure. GO term analysis was next conducted and significant (*p* < 0.05) GO terms were plotted (Fig. 6c). In both HC Chronic and IVSA, processes related to gene regulation and synaptic signaling were impacted. In the Conditioned group, networks related to mitochondrial activity and metabolic activity were affected. The most connected genes or “hub genes” (defined in the supplemental methods section) were also identified from the top module of each networks and the top 30 were plotted (Fig. 6d) [20, 51]. Overall, these analyses provide evidence for the specific cell types, biological processes and genes that cocaine alters within the VTA.

## Discussion

These data provide the first unbiased assessment of genome-wide gene expression and gene networks in the VTA across several commonly employed cocaine exposure paradigms for studying cocaine-associated processes. We analyzed these data to identify the key genes and biological processes that are engaged by various cocaine exposure paradigms. In addition, we found novel predicted upstream transcription regulators of cocaine-induced changes in gene expression and gene networks within the VTA. Lastly, this study used individual variability from cocaine self-administration to identify gene expression changes that correlated with behavioral measures related to cocaine intake.

From examining broad patterns of gene expression, we found that the more complex the cocaine exposure paradigm (perhaps where more novel cocaine-related stimuli are experienced) the more complex gene expression patterns become within the VTA. As may be predicted, we found that each paradigm largely induces specific molecular mechanisms within the VTA. Our genome-wide results are supported by previous studies examining specific genes (Choi et al., 2011; Cameron et al., 2014). Two important caveats of the study are that we only examined males and at only one time point. Future studies should examine whether sex-dependent and/or estrus cycle-dependent effects on transcription occur within these paradigms [52, 53], and different time points, as cocaine is known to differentially affect gene expression in a time-dependent manner [3, 22, 54]. It is also important to consider the variability in cocaine dosing within IVSA mice, as mice did not consistently self-administer 20 mg/kg dose in comparison to the other paradigms. Lastly, we did not examine protein levels, which is an additional caveat in the interpretation of these data.

Despite observing paradigm-specific patterns of gene expression, we still found overlapping DEGs across paradigms. These include several DEGs that were previously reported, (e.g., *Fos*, *Nr4a1/2, Cartpt, Bdnf*) and genes not previously implicated in cocaine action, including *Maff, Fgf20*, *P2ry13, Pet117, Entpd2, Kcnab1, Nefl/h*. These genes have reported roles in one of the following activities: regulating activity-dependent changes in gene expression [39], neuroprotection [55], neurogenesis, energy regulation [41], potassium channel activity [56], and axon structure [57]. Among these DEGs, *Maff* is reported to have sex-specific expression profiles in the rat prefrontal cortex [58], therefore it may play a pivotal role in sex specific cocaine-induced changes in the VTA. These transcripts and processes serve as potential targets for understanding the mechanisms of cocaine-seeking and for therapeutics of cocaine addiction.

To better understand the function of these novel targets, we examined the biological processes associated with these genes across the different paradigms. Given the few biological processes affected by acute cocaine in comparison to chronic cocaine, it is possible repeated cocaine exposure dysregulates more cellular processes, including energy regulation/metabolism. In addition, consistent with other findings, we found more changes in gene expression related to post synaptic and cytoskeletal structures occur within cocaine learning-related paradigms (e.g., IVSA and Conditioned) [59, 60]. There were also instances where GO terms were shared across paradigms, however distinct DEGs were identified. This suggests that cocaine may affect similar biological processes, yet the upstream signaling/genes that control these mechanisms may be paradigm specific. Several processes related to ion channel activity altered by Conditioned and IVSA cocaine paradigms were found in our datasets. Although cocaine affects intrinsic excitability in various brain regions [61, 62], few studies have examined altered expression of ion-channel related genes [63, 64]. In regard to IVSA cocaine, circadian regulation of gene expression was among the processes affected only by this paradigm, with changes in *Per1/2/3*, *Cry1/2*, and *BHLHE40*. This may be indicative of the altered circadian regulation of dopamine following cocaine self-administration [65, 66]. When comparing chronic cocaine paradigms, we find that altered expression of genes related to mitochondrial function following HC Chronic and IVSA cocaine. This supports recent findings where chronic cocaine alters mitochondrial function in the mouse striatum and in the hippocampus of cocaine-addicted individuals [67, 68]. Overall, our analysis sheds light on the biological processes affected by a particular cocaine exposure paradigm/aspect of cocaine-seeking (i.e., cocaine-associated memories vs. cocaine reinforcement) as well as processes shared across multiple paradigms, which may be critical for promoting general cocaine action.

Several upstream regulators of cocaine-induced changes in gene expression were identified from our analysis. These include TFs that were unique to specific paradigms, as well as regulators associated with all cocaine paradigms. This suggests that despite there being distinct changes in gene expression specific to a paradigm, there are perhaps master regulators of cocaine-induced transcription affected by cocaine regardless of the paradigm. We also identified upstream regulators that may have sex-specific roles in the VTA. For example, pharmacological studies suggest that ER-alpha activity, one upstream regulator we identified from both HC Chronic and IVSA samples, is protective against the effects of cocaine in males, but promotes cocaine action in females [69]. Examining the putative downstream targets of ER-alpha that we identified in our datasets within both males and females may provide new insight into the sex-dependent roles of ER-alpha in response to cocaine. Overall, the identification of novel TFs provides new leads to pursue in understanding cocaine action in specific paradigms and across paradigms.

We also found that the expression levels of several genes correlate with behavioral measures of cocaine IVSA. This includes *Mef2a*, *Zbtb16* and *Hip1*, which are DEGs also identified in previous studies [3, 22]. In terms of potential functional roles, ZBTB16 interacts with histone deacetylases like HDAC1 and HDAC5 [70], which are mechanisms known to be dysregulated by cocaine [71]. HIP1 is an endocytic adaptor protein that plays a role in endocytosis and internalization of AMPA receptors [72]. Cocaine has shown to affect synaptic levels of AMPAR in the VTA [5], thus HIP1 could be a potential target for regulating these mechanisms. This correlational analysis (IVSA behavior-gene expression) we applied to our data potentially reveals genes critical for cocaine-seeking behaviors.

From WGCNA, we identified gene networks and cell-types within the VTA that are altered by cocaine across exposure paradigms. Gene networks within glial cells, particularly astrocytes, are affected by both chronic cocaine paradigms. This supports research showing that astrocytes and microglia are affected by cocaine exposure and drive synaptic processes [73–75]. Similar to our DEG GO term analysis, biological processes affected by cocaine-altered gene networks are implicated in mitochondrial and synaptic processes. Additional processes identified from WGCNA were related to gene regulation. This is consistent with recent work examining how cocaine alters RNA splicing to promote cocaine seeking [76], and also introduces other processes for the field to examine (i.e., ribosomal and viral-like transcription). Lastly, our hub gene analysis is in agreement with our DEG analysis; distinct gene sets are dysregulated by cocaine in paradigm-specific manner, however they result in similarly affected biological processes. This underscores the importance of these identified hub genes and calls for future studies to examine their relevance in a paradigm-specific manner.

We present here novel (and confirm previously identified) candidate genes, biological processes, and transcriptional regulators that may underlie cocaine-induced plasticity within the VTA. It will be critical to, in follow-up studies, validate these findings with proteomics and genetic manipulations of the identified DEGs, hub genes and upstream regulators. These results underscore the importance of choosing a cocaine exposure paradigm, as distinct effects on molecular mechanisms in the VTA are seen from paradigm to paradigm. We also highlighted several genes of interest and transcriptional regulators of unique aspects of cocaine exposure in the VTA. As such this is a unique resource, as it examines how cocaine affects transcription within the VTA across several classic cocaine exposure paradigms that are used within the field. Overall, these data provide a thorough analysis in identifying the molecular mechanisms affected within the initial stages of cocaine action.

## Funding and disclosure

This work was supported by funding from the National Institutes of Health (F31 DA048527 to RRC; R01 DA047981 and DA025922 to MAW; R01 GM123558 to PB). The authors declare no competing interests.

## Supplementary information

Supplemental Figures and Methods

Supplemental Data Table 1

Supplemental Data Table 2

Supplemental Data Table 3

Supplemental Data Table 4

Supplemental Data Table 5
