# Supplementary material for: Cocaine induces paradigm-specific changes to the transcriptome within the ventral tegmental area
Source: Neuropsychopharmacology. 2021 Jun 21;46(10):1768–79. doi: 10.1038/s41386-021-01031-4 (PMC8357835; doi:10.1038/s41386-021-01031-4)
Supplement: Supplementary file 1 — Supplemental Figures and Methods [file 41386_2021_1031_MOESM1_ESM.pdf]

Supplemental Figures.

Supplemental Figure 1.

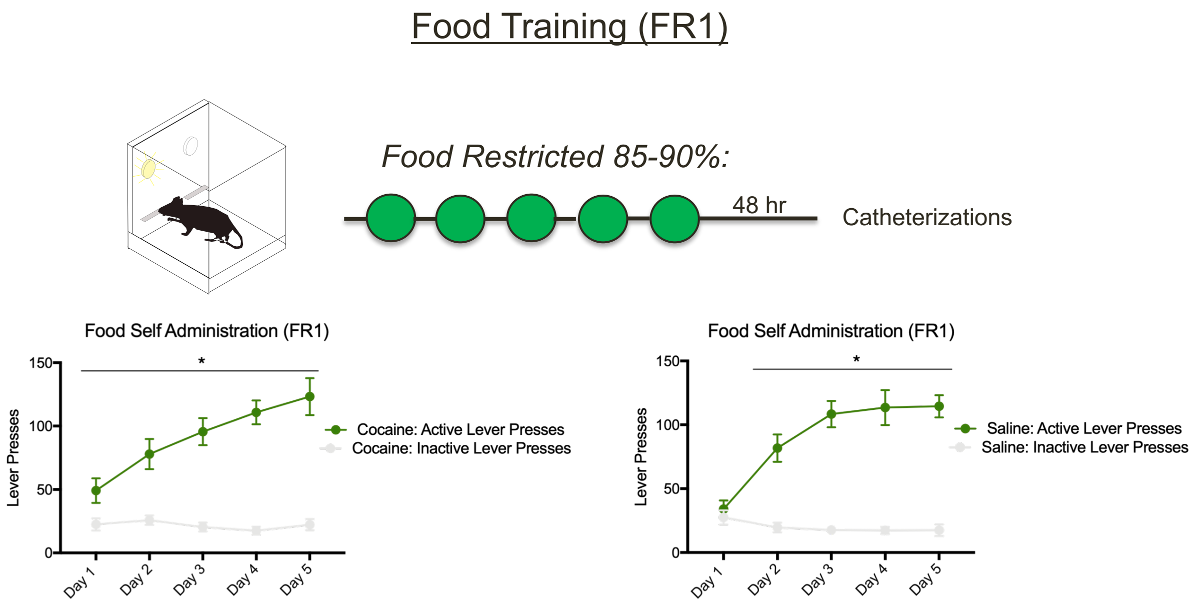

Supplemental Figure 1. FR1 Operant Food Training for Cocand Saline IVSA mice. a. While mildly food restricted, mice underwent food training at an FR1 schedule for 5 days. b. Mice that were later assigned to undergo cocaine self-administration acquired operant food training (Two-way ANOVA repeated measure Lever effect (F1,14)=106.4, p<0.0001; Session effect F(4,56)=9.091, p<0.0001; Session x Levers Interaction effect F(4,56)=13.49, p<0.0001). c. Mice that were later assigned to undergo saline self-administration acquired operant food training (Two-way ANOVA repeated measure Lever effect (F1,7)=70.7, p<0.0001; Session effect F(4,28)=10.86, p<0.0001; Session x Levers Interaction effect F(4,28)=23.14, p<0.0001).

Supplemental Figure 2.

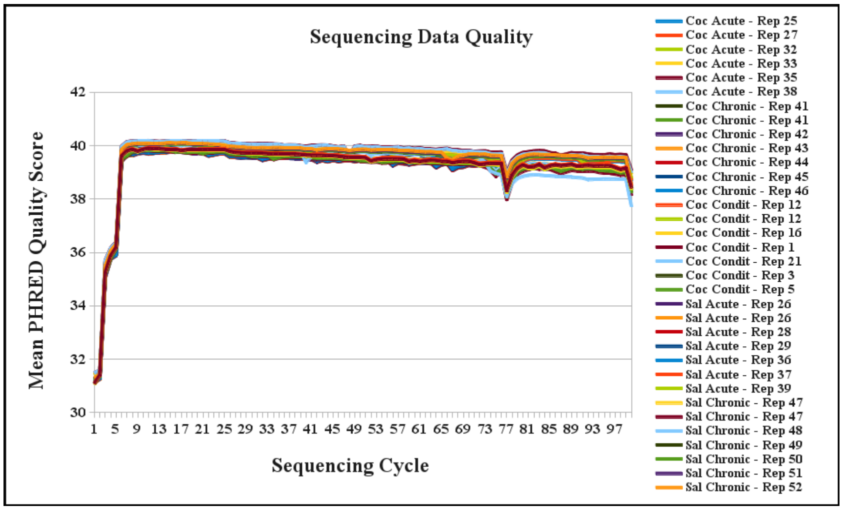

Supplemental Figure 2. Mean PHRED quality scores for each animal.

Supplemental Figure 3.

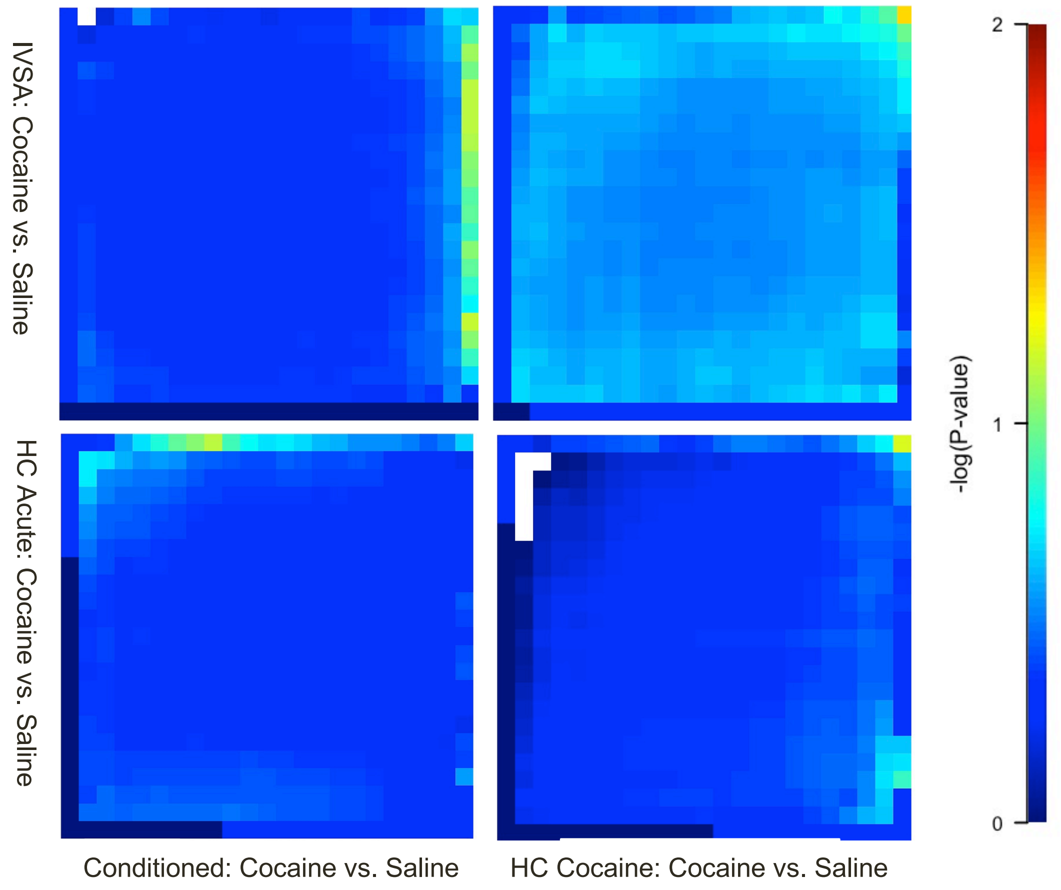

Supplemental Figure 3. Cocaine induces distinct paradigm-specific changes to the VTA transcriptome. Using threshold-free comparison of DEGs by rank-rank hypergeometric overlap, little overlap in DEGs was found between cocaine paradigms. Pixels represent the overlap between the transcriptome of each comparison as noted, with the significance of overlap (−log10(p-value) of a hypergeometric test) color coded.

Supplemental Figure 4.

Fisher's Exact Test

Figure	overlap	left	right		background1	p-val1		background2	p-val2
2a	3	27	76		15000	0.000515		181	1.97E-05
2b	1	19	55		15000	0.0626		181	4.20E-03
2c	1	29	85		15000	0.1586		261	4.68E-05
2d	3	17	126		15000	0.00051		261	1.04E-03
2e	6	73	229		15000	0.00148		513	1.68E-15
2f	5	51	149		15000	0.000171		513	7.21E-05
2g	21	65	214		15000	1.18E-19		579	5.45E-04
2h	4	125	150		15000	0.0408		579	6.99E-15

Background 1, p-val1. Testing all genes from the RNA-seq analysis after filtering out low gene expressions: the number of background genes used is 15000 (the last cell in the contingency table).

Background 2, p-val2. Testing only the DEGs in two groups: for example in CPP VS IVSA, the total number of DEGs from both groups is 579, then 579 is the number of background genes used (the last cell in the contingency table).

Supplemental Figure 5.

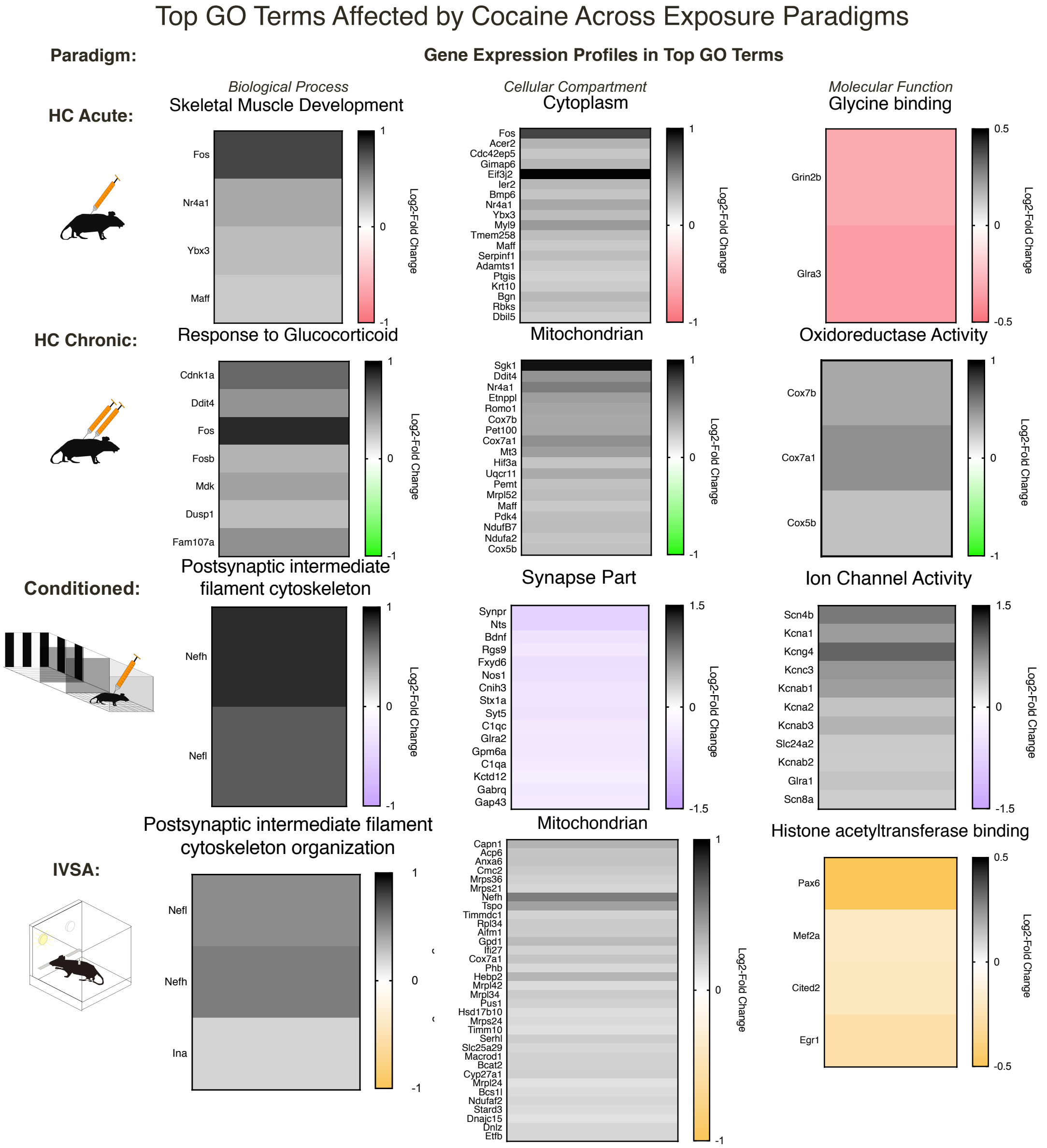

Supplemental Figure 5. **Predicted biological processes altered by each cocaine paradigm.** Top gene ontology (GO) terms of both upregulated and downregulated DEGs within each paradigm are listed (left). Heat maps displaying the log-fold changes of DEGs associated with a particular GO term are shown (right).

Supplemental Figure 6.

Common Predicted Upstream Regulators of Cocaine-induced Gene Expression

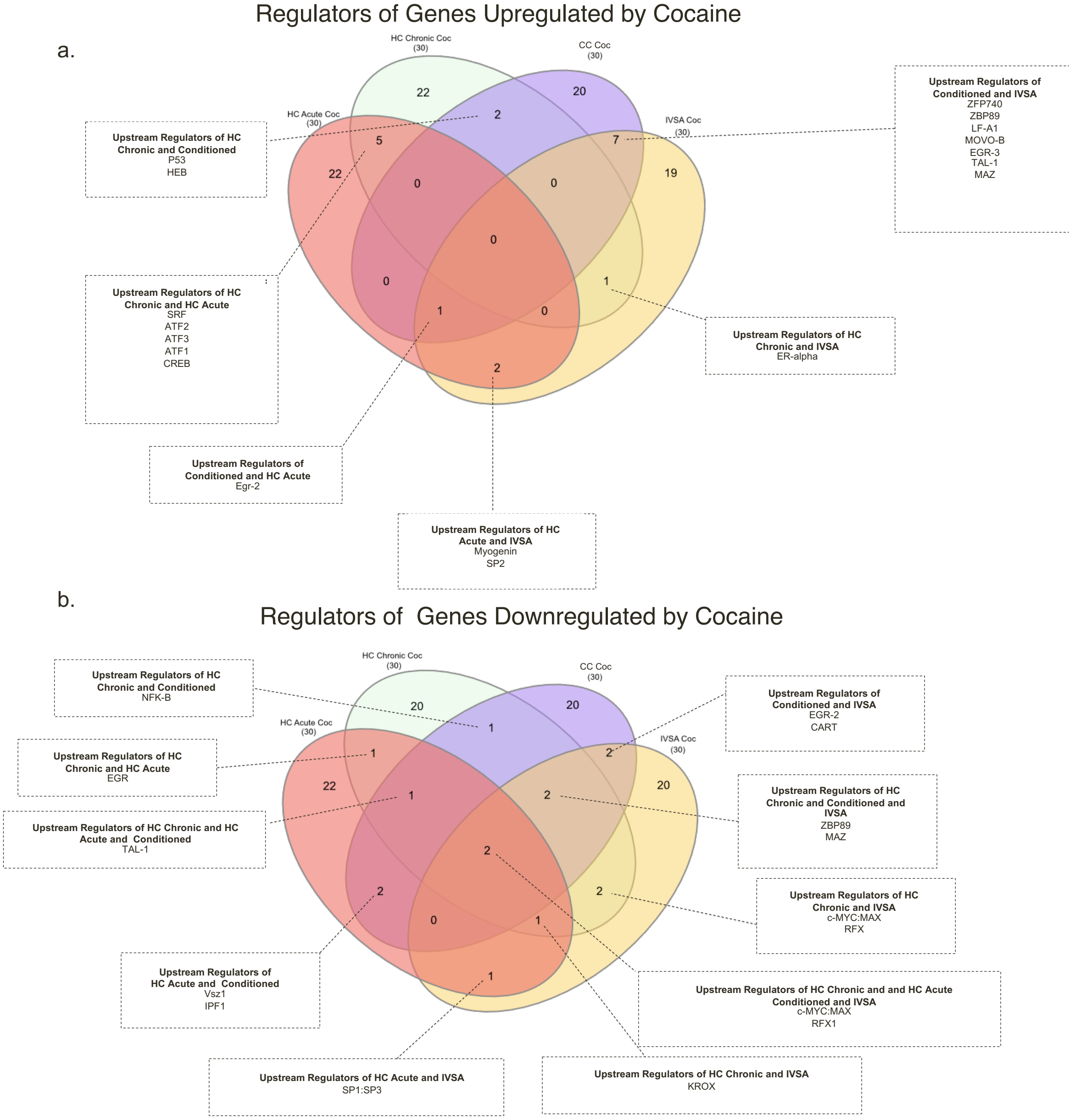

Supplemental Fig 6. **Common predicted upstream regulators of both upregulated genes and downregulated genes induced by cocaine in each paradigm.** a. Upstream regulators of genes upregulated by cocaine. There were no common upstream regulators of genes upregulated by cocaine across all paradigm from the top 30 regulators. However, several common upstream regulators were found between paradigms. b. Upstream regulators of genes downregulated by cocaine. Common upstream regulators of genes downregulated by cocaine across all paradigms include cMyc:Max and RFX1.

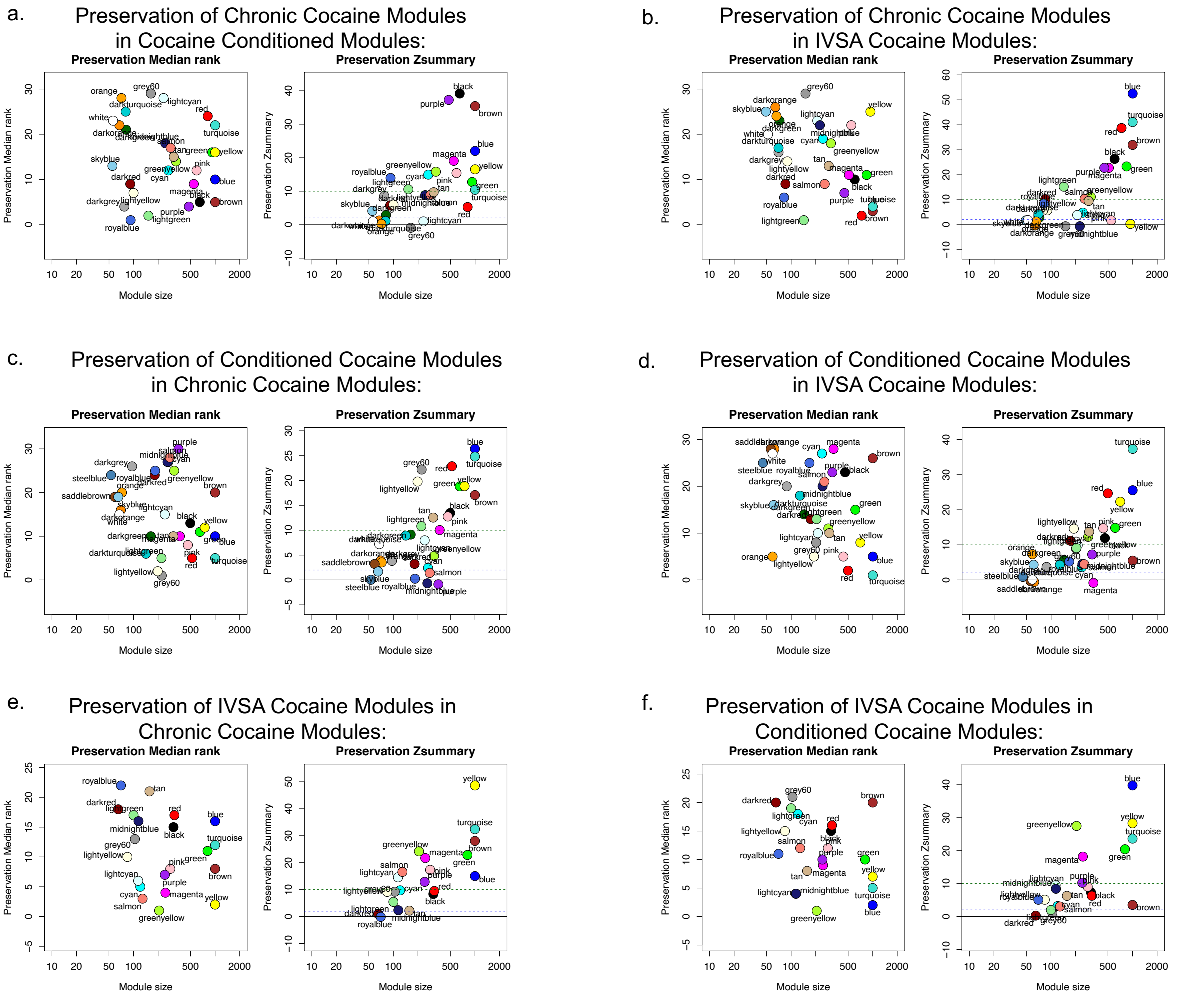

Supplementary Figure 7. **Preservation of VTA WGCNA modules across cocaine paradigms.** A-f. Modules from each cocaine paradigm are compared across paradigms. Median rank graphs show the modules with stronger observation preservation (modules with lower median rank). In the preservation summary graphs, dotted green and blue lines are thresholds for preservation (preserved: Zsummary>10 ; non preserved: Zsummary<2), which strongest preservation having higher Zsummary scores.

Supplemental Methods.

RNA Sequencing:

RNA was isolated from the ventral tegmental area punches, using the RNeasy minikit (Qiagen, 74104). RNA quality was assessed by Bioanalyzer and only samples with an RNA integrity number greater than 9 were included in our analysis. cDNA libraries for all groups were prepared using the TruSeq RNA Sample Preparation Kit (Illumina, RS-122-2001). One hundred nanograms of total RNA from each mouse was purified with poly-T oligo-attached magnetic beads and heat fragmented. First and second strands of CDNA were synthesized and then purified. After blunting of the ends occurred, the 3′ end was adenylated to prevent concatenation of the template during adapter ligation. For each treatment group, a unique adapter set was added to the ends of the cDNA and the

libraries were amplified using PCR. The quality of the library was assessed by Bioanalyzer and quantified using qRT-PCR with a standard curve prepared from a commercial sequencing library (Illumina). Samples were mutliplexed, with each treatment group represented in each flow cell of the sequencer. 10 nM of each library was pooled in four multiplex libraries and sequenced on an Illumina HiSeq 2500 instrument during a single-read 100 bp sequencing, run by the Genomic High-Throughput Facility at the University of California, Irvine. The resulting sequencing data for each library were post-processed to produce FastQ files. The data were then demultiplexed and filtered using Illumina CASAVA 1.8.2 software as well as in-house software. Control reads successfully aligned to the PhiX control genome or poor quality reads failing Illumina's standard quality tests were removed from the following analyses. The quality of the remaining sequences was further assessed using PHRED quality scores produced in real time during the base-calling step of the sequencing run.

Alignment to the reference genome and transcriptome

The reads from each experiment were separately aligned to the mm10 reference genome using Bowtie2. The first 10 bases from the start of the read are trimmed using Trimmomatic, and reads shorter than 50 bp are excluded. Reads uniquely aligned by both tools to known exons or splice junctions with no more than two mismatches on any 25 bp fragment of the read were included in the transcriptome. Similarly, reads matching several locations in the reference genome were removed from analysis. The percentage of reads assigned to the reference genome and transcriptome using this protocol is reported for each group of replicates (**Supp. Data 4)**. This resulted in an average of approximately 53 million reads per sample.

Downstream DEG analysis:

Top up- or down-regulated genes are identified for further analysis. This includes identification of differentially expressed upstream transcription factors and their downstream targets using transcription factor binding site (TFBS) enrichment analysis of all promoters using the MotifMap database. In MotifMap, the default parameters of 10,000 bp upstream and 2,000 bp downstream were used. Gprofiler was used to identify Gene Ontology terms from DEGs. This rank-rank hypergeometric overlap (RRHO) plots(Raudvere et al. 2019). All RNA-seq files are available on Gene Expression Ominbus (GEO GSE155313).

Significance of Overlapping Genes:

We performed Fisher exact test (FET) to calculate the significance in overlapping genes in Figure 2a-2h. Two sets of FET were ran using different background genes in the last cell of the contingency table: (1) we included roughly 15000 genes as background genes and those genes were obtained by filtering out genes with low FPKM values. The FET statistics from this set of background genes were shown in p-val1 in the table below. (2) we included only the DEGs relevant to the groups in comparison (for example, the background genes used in Figure 2a are DEGs in HC Acute plus DEGs in HC Chronic). The number of background genes used for FET and the FET statistics were shown in

background2 and p-val2 in the table. By using the first set of background genes described in (1), most of the overlaps were significant except for 2b and 2c. By using the other set of background genes described in (2), the large p-values were improved and overlapping genes in all groups were significant.

WGCNA analysis:

Co-expression network analysis was performed using the R WGCNA library. The soft thresholding power beta was chosen for each paradigm based on the criterion of approximate scale-free topology. By using hierarchical clustering on the dissimilarity in the topological overlap matrix (dissTOM = 1-TOM), gene modules were calculated for each paradigm. Module eigengenes, 1st principle component of the module, were computed. GO term enrichment analysis was performed on top three modules with the most genes using R library enrichr. The top modules enriched from each paradigm were calculated using the FET statistics of over-representation of differential genes (DEGs) in each module. We selected a top modules that showed the highest significant enrichment (p-value<0.05) after FDR correction. For each paradigm, the top 30 hub genes were identified and visualization of the top 30 hub genes were shown as network graphs plotted by Cytoscape(Otasek et al. 2019). Using the R function softconnectiviity, we calculated the kME, which was used for calculating intra-modular connectivity for every gene. We took the top 30 most connected genes within a module and labeled them hub genes. For the WGNCA enrichment, we used DEGs that were detected from each paradigm to determine whether that same paradigm had modules enriched for their own DEGs (i.e. DEGs from IVSA enriched in the IVSA WGCNA).

Cell-type enrichment analysis was performed on the top five modules in each paradigm. Cell-type specific marker genes for six cell types in the midbrain include: astrocyte, cholinergic, dopaminergic, microglia, oligo, and serotenergic(Mancarci et al. 2017). P-value from Fisher exact test was calculated and corrected by FDR multi-test correction.

qPCR: RT-qPCR was performed as described previously. cDNA was created using the Transcriptor First Strand cDNA Synthesis kit (Roche Applied Science). Primers were designed using the Roche Universal Probe Library (**Supp. Data 5**). All values were normalized to Hprt5 expression levels. Analyses and statistics were performed using the Roche and REST 2009 software based on the Pfaffl method.

Statistical Analysis for Behavior.
Prism was used for all graphing and statistical analysis of behavior. Significance thresholds were set at $p < 0.05$. Main effects and interactions were analyzed by either a one-way ANOVA (CPP data) or two-way ANOVA repeated measures (IVSA data). Tukey post-hoc comparisons were performed when appropriate. All analyses are two-tailed, with an α value of 0.05 required for significance. Error bars in all figures represent SEM. For all experiments, values ± 2SD from the group mean were considered outliers and were removed from analyses.

Differential Gene Expression using Linear Regressions

In a supplementary table (Supp. Table 1), we listed DEGs identified from our samples using DESeq2 linear regression analysis, as performed in(Swarup et al. 2019; Swarup et al. 2020). Briefly, gene expression levels were first quantified for all the samples using union gene models with HTSeq-Counts (v0.5.4). If genes were expressed in 80% of the samples with >10 reads, then they were included in analysis. Read counts were then normalized for library size using cpm function of the edgeR package in R to obtain fragments per kilobase million mapped reads (FPKM) values. Log2 transformation followed by linear regression was performed to obtain DEG_LR lists.

Bibliography

Mancarci, B.O., Toker, L., Tripathy, S.J., et al. 2017. Cross-Laboratory Analysis of Brain Cell Type Transcriptomes with Applications to Interpretation of Bulk Tissue Data. *eNeuro* 4(6).

Otasek, D., Morris, J.H., Bouças, J., Pico, A.R. and Demchak, B. 2019. Cytoscape Automation: empowering workflow-based network analysis. *Genome Biology* 20(1), p. 185.

Raudvere, U., Kolberg, L., Kuzmin, I., et al. 2019. g:Profiler: a web server for functional enrichment analysis and conversions of gene lists (2019 update). *Nucleic Acids Research* 47(W1), pp. W191–W198.

Swarup, V., Chang, T.S., Duong, D.M., et al. 2020. Identification of conserved proteomic networks in neurodegenerative dementia. *Cell reports* 31(12), p. 107807.

Swarup, V., Hinz, F.I., Rexach, J.E., et al. 2019. Identification of evolutionarily conserved gene networks mediating neurodegenerative dementia. *Nature Medicine* 25(1), pp. 152–164.
